# Use of Honey Bees and Hive Products as Bioindicators to Assess Environmental Contamination in Targeted Areas of the Campania Region (Italy)

**DOI:** 10.3390/ani14101446

**Published:** 2024-05-13

**Authors:** Patrizio Catalano, Francesco Della Sala, Maria Cavaliere, Carla Caputo, Domenico Pecoraro, Giulia Crispino, Stefania Lettera, Giulia Caioni, Mauro Esposito, Antonio Verre, Luigi Castellone, Enrico Bianco, Michele Amorena

**Affiliations:** 1 Department of Prevention of ASL Napoli2nord, Frattamaggiore, 80027 Naples, Italy; patrcatalano@libero.it (P.C.); maria.cavaliere@aslnapoli2nord.it (M.C.); carla.caputo@aslnapoli2nord.it (C.C.); domenico.pecoraro@aslnapoli2nord.it (D.P.); giulia.crispino@aslnapoli2nord.it (G.C.); stefania.lettera@aslnapoli2nord.it (S.L.); antonio.verre@aslnapoli2nord.it (A.V.); luigi.castellone@aslnapoli2nord.it (L.C.); enrico.bianco@aslnapoli2nord.it (E.B.); 2 HSE Manager of Campania A2A Ambiente S.p.A, Acerra, 80011 Naples, Italy; francesco.dellasala@a2a.it; 3 Department of Bioscience and Agro-Food and Environmental Technology, University of Teramo, 64100 Teramo, Italy; mamorena@unite.it; 4 Centro di Referenza Nazionale per l’Analisi e Studio di Correlazione tra Ambiente, Animale e Uomo, IZS Mezzogiorno, Portici, 80055 Naples, Italy; mauro.esposito@izsmportici.it

**Keywords:** bioindicators, honey bees, hive products, honey, wax, waste-to-energy plant, pollutants

## Abstract

**Simple Summary:**

Assessing the environmental quality of specific geographical areas, particularly those in proximity to industrial zones or the sites of waste-to-energy plant constructions, emerges as a critical concern in contemporary environmental science. The use of biological indicators, such as bees, represents a noninvasive methodology for environmental monitoring. By taking advantage of the extension of their foraging radius and evaluating the quality of the hive’s products, it is possible to obtain an in-depth snapshot of the environmental state of the surrounding area. In the context of our study, the vicinity of the Acerra waste-to-energy plant was first evaluated using bees as bioindicators, thereby aiming to detect the potential presence of hazardous waste combustion byproducts. Following promising initial results, it was decided to look for additional pollutants (such as pesticides, metals, and polycyclic aromatic hydrocarbons) and establish a second apiary in the Caivano area to further affirm the bees’ efficacy in pinpointing specific geographic zones impacted by anthropogenic activities. Research focusing on bees and apiary products underscored the heightened sensitivity of this bioindicator to various environmental pollutants, whose presence delineated the anthropogenic utilization of the studied area.

**Abstract:**

In recent years, biomonitoring has gained more attention, particularly when assessing the environmental health of significant areas, such as those near waste-to-energy facilities. These requirements coincide with the chance to detect environmental pollutants using sensitive organisms. Bees were shown to be quite effective in evaluating the presence of certain compounds by analyzing their associated matrices, such as pollen, honey, or wax. In our study, we employed the honey bee (*Apis mellifera*) as an indicator to initially monitor the vicinity of the waste-to-energy plant in Acerra, which is situated in the Campania region of Italy. The primary aim was to determine whether the facility was accountable for any environmental releases of dioxins or dioxin-like compounds. Then, we assessed the presence of additional pollutants in the same area, including trace elements, polycyclic aromatic hydrocarbons, and pesticides, released by human activities. To obtain further information about environmental quality, a second biomonitoring station was installed near the Caivano S.T.I.R. (Waste Shredding, Sifting, and Packaging Plant). The results showed the dioxin levels did not exceed predetermined limitations at the Acerra site, thus demonstrating the efficacy of the waste-to-energy facility and the bees’ ability to detect the presence of other pollutants. Additionally, this biomonitoring system exhibited sensitivity to environmental variations, thereby enabling the evaluation of xenobiotic flux between two proximate zones and across temporal scales. This pioneering study suggests the advantages of utilizing bees to detect a wide range of contaminants, thereby providing valuable insights into environmental quality and potential health risks for both ecosystems and human populations.

## 1. Introduction

Biotic systems, such as arthropods, can be employed to evaluate the environmental impacts of human activity and the existence of some biogeographic alterations [1,2]. The Hymenoptera order has acquired great relevance in this regard [3,4,5], and honey bees (*Apis mellifera*) have long been used as indicators of chemical pollution [6,7,8,9]. Compared to the difficulties of conducting direct sampling, they guarantee a greater volume and thoroughness of data. They are gregarious insects that travel over distances of many kilometers when foraging [10], thus putting them in contact with a variety of xenobiotics. Thus, it is possible to assess the presence of a wide range of chemicals by performing analyses on different matrices, which include pollen, bees, honey, and beeswax [11,12,13,14]. Beeswax represents a true memory of the beehive and can linger there for a long time, thereby causing liposoluble contaminants to accumulate. For these reasons, it allows for the detection of substances that have not been used recently or are present in greater amounts than pollen or bees [15]. Honey is another matrix of relevance in the assessment of the impacts of human activity. It is a valuable tool for tracking the presence of heavy metals, and there are several examples of its usage as a bioindicator in the literature [16].

A growing number of published papers emphasize the significance of biomonitoring in ensuring human health, thus also indicating the success of using bees and their products [7,9,17,18,19,20]. Bees are an effective resource not only for learning about the state of a particular region but also, most importantly, for highlighting discrepancies between two adjacent areas to retrace the flow and movement of xenobiotics.

The innovative use of bees as bioindicators may involve monitoring areas near facilities related to urban waste treatment, particularly in critical areas like the Campania region in Italy [21]. Situated on the outskirts of Acerra, the waste-to-energy (WtE) facility is close to both rural and industrial regions. Active since 2009 and with an installed capacity of 107.5 MWe, the plant has been certified ISO 9001 [22], ISO 14001 [23], EMAS, and ISO 45001 [24]. The unit includes three combustion lines and uses residual municipal waste, recovering unsorted solid waste pretreated in the Caivano S.T.I.R. (Waste Shredding, Sifting, and Packaging Plant) of the Campania region. Considered one of the most avant-garde plants in Europe, it was designed and built using the best technologies to ensure maximum environmental protection, and it is continuously monitored both to guarantee its correct functioning and to maintain the emission values well below the established thresholds by the European Directive (2010/75/EU) and Italian law (legislative decree n. 152/2006 and 133/2005). One of the major concerns regarding the installation of WtE plants involves the potential production and release of polychlorinated dibenzo-p-dioxins (PCDDs), polychlorinated dibenzofurans (PCDFs), and related compounds that include polychlorinated biphenyls (PCBs) [25]. Gathering information on this aspect requires the use of an integrated approach starting from the verification of the main emission sources to monitoring air quality in surrounding areas and conducting model simulations [25,26]. As part of this study, an environmental investigation apparatus based on the use of bees as bioindicators was developed to concurrently evaluate the performance of the WtE plant and the applicability of this kind of bioindicator. In the first phase (carried out from 2019 to 2020), we focused on examining the levels of PCDDs, PCDFs, and PCBs using an apiary located near the WtE plant. The analyses were conducted on the wax, as it is the appropriate matrix for assessing liposoluble substances. Having obtained reassuring results, additional environmental pollutants such as pesticides, metals, and polycyclic aromatic hydrocarbons (PAHs) were investigated in Acerra. The detection of pollutants continued in 2021 and 2022, and, knowing that another point of interest exists a few kilometers from Acerra, a second apiary was established near the S.T.I.R. facility in Caivano in 2021. This area is characterized by significant industrial activity, and for this reason, we wanted to verify whether the bee bioindicator could highlight the different land uses of this territory, which are characterized by the presence of industries, vehicular traffic, heating, agricultural activities, solvent use, and fuel distribution. Although this is a preliminary study that requires further integrations, it demonstrated the possibility to monitor critical areas through the use of bees and related matrices, thus also representing a starting point to create a sensitive system which provides reliable information.

## 2. Materials and Methods

### 2.1. Area of Interest

**Figure 1:**
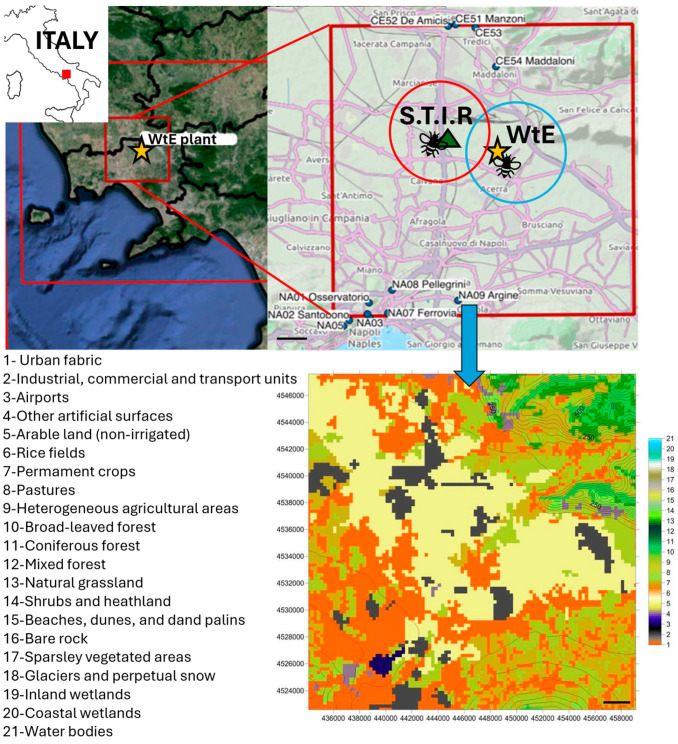
Map of the area of interest. The location is indicated as a red square on the small-size map of Italy in the top left corner. The monitoring stations in Acerra (coordinates: 40.94411, 14.37144) and Caivano (coordinates: 40.98981, 14.30229) are situated in the Campania region in the vicinity of the waste-to-energy plant (indicated by the yellow star), and the S.T.I.R (green triangle), respectively. The bee icons indicate the apiaries, and the foraging radii of the honeybees are indicated by the red and blue circles, which correspond to 3 km. Below that is the representation of the intended use of the territory under study (horizontal resolution: 250 m), with land use codes (21-class classification) reported on the left. Scale bars: 2000 m (2 km). Abbreviations: WtE: waste-to-energy plant; S.T.I.R.: Waste Shredding, Sifting, and Packaging Plant.

The geographical area of focus is reported in Figure 1 and is situated in the Campania region (Italy) at the juncture of the provinces of Naples and Caserta spanning the plain adjacent to the boundaries of the Naples metropolitan region. It extends eastward to the vicinity of Nola and encompasses a segment of the Aversa rural region to the northwest. The intended use of the territory under study is represented in the bottom right corner of Figure 1. Land use codes are defined according to a 21-class classification—derived from the 44-class European standard CORINE Land Cover—(EEA Data Service, http://www.eea.europa.eu/data-and-maps (accessed on 3 March 2019)) by merging some categories and according to the text shown next to the map. The presence of urbanized areas (Acerra in the center, the metropolitan area of Naples to the south, Caserta and Maddaloni to the north) in orange, the industrial areas in gray, and the predominantly agricultural area in the central part of the domain are evident. The Naples Capodichino airport to the southwest is also recognized in purple, as well as the location of the quarries (in purple) on the reliefs in the northeastern quadrant. The area near Caivano is more rural, and heavy vehicular traffic prevails.

The apiaries were localized in two different sites, Acerra and Caivano, as reported in the map (Figure 1). Specifically, the geographical coordinates for the apiary in Acerra are latitude 40.94411 and longitude 14.371, while the latitude and longitude of the second apiary in Caivano are 40.98981 and 14.30229.

### 2.2. Sample Collection

The research was conducted from 2019 to 2022 at the Acerra monitoring station near the WtE facility and from 2021 to 2022 at the monitoring station at the S.T.I.R. company in Caivano. In each apiary, three hives (colonies) were identified and selected for sample collection. During the initial phase of the study at the Acerra apiary from 2019 to 2020, as part of the dioxin assessment, 50 g of wax from each hive were collected and then combined to form a pool for analysis. Sampling was conducted in June, July, and August for 2019 and in May, July, August, and September for 2020.

To analyze trace elements (metals), pesticides, and polycyclic aromatic hydrocarbons (PAHs) in pollen, honey, and beeswax at both of the monitoring stations, sampling was carried out at the beginning and end of each beekeeping season for each study year, with three hives sampled at each location. As for the research on PAHs in bees, sampling was conducted monthly from June to September for each year.

The study involved sampling during the period from early June (which corresponds to the first honey harvest) to October. Each apiary consists of 3 colonies, with a foraging radius of about 3 km. The sampling conducted in Acerra in 2019 and 2020 (as part of the assessment of dioxins in the wax) was monthly (a sampling was also performed in May 2020), but only one sample was taken from each of the three hives, and the results were pooled. In the other cases, two samplings were performed each year on three hives.

#### 2.2.1. Pollen

To collect the pollen, a special trap was placed at the entrance to the hive, forcing the forager bee to return to the hive through a grid with holes of a calibrated diameter. The collected pollen grains were automatically unloaded and deposited into a drawer below. Pollen samples, of at least 10 g per station, were frozen at −18 °C and then sent to the laboratory for analysis.

#### 2.2.2. Honey Bees

The sample included approximately 250 bees, which were collected in special containers without the use of a smoker. After being frozen, the bees were delivered to the laboratory. Each sample was labelled with the hive’s identifying number, the sample code, and a copy of the sampling report.

#### 2.2.3. Honey

The fresh honey (with a humidity of >18%) was collected directly from the uncapped honeycombs, and stored at 4 °C until the beginning of the analytical procedure.

#### 2.2.4. Honey Wax

At the beginning of the monitoring, empty frames were placed in the hives to allow the bees to build on-site the wax that would be used for the detection of the analytes. The material was frozen at the time of the collection and stored at a temperature of −18 °C until it arrived at the lab.

### 2.3. Analysis

#### 2.3.1. Dioxins and Dioxin-Like Polychlorinated Biphenyls

Each sample (50 g of honey wax) was kept at −20 °C for 24 h before being homogenized with liquid nitrogen using a crushing mill (IKA, Wilmington, NC) and examined for the presence of dioxins PCDD/PCDFs and PCBs (Table S1). The determination of PCDD/Fs and PCBs was carried out using analytical techniques based on global standards for dioxin analysis, such as EPA 1613 [27], as well as in compliance with relevant European Directives. 

Samples of beeswax were immediately dissolved in 20 milliliters of n-hexane. All solvents were trace analysis grade (Carlo Erba Reagents, Val-de-Reuil, France). A 4 cm diameter multilayer column comprising (top to bottom) Na_2_SO_4_, 44% H_2_SO_4_/silica, 22% H_2_SO_4_/silica, NaOH/silica, and AgNO_3_/silica was used to purify the sample extracts. N-hexane was used to elute PCDD/Fs. The refined extracts underwent fractionation within SUPELCO (Bellefonte, PA, USA) prepacked carbon tubes (Supelclean Envi-Carb). Following the evaporation of the resulting PCDD/ SF fractions to 15 μL under a nitrogen stream, the corresponding PCDD/F ^13^C syringe standards (1,2,3,4-TeCDD and 1,2,3,7,8,9-HxCDD) were added. Samples were examined using an Autospec Ultima high-resolution mass spectrometer (Micromass, Manchester, UK) connected to an Agilent 6890N gas chromatograph (Santa Clara, CA, USA) running at 10,000 resolving power and in electronic impact ionization mode. Samples were injected (2 μL) into the injector at 280 °C for 1 min in splitless mode to perform the PCDD/F analysis. A Restek (Bellefonte, PA, USA) RTX-5MS column (60 m × 0.25 mm × 0.25 μm) was used to fit the chromatograph. Helium in constant pressure mode at 250 kPa served as the carrier gas. Temperatures were set to 150 °C for one minute and then increased at 30 °C/min to 200 °C, 3 °C/min to 235 °C (kept for ten minutes), and 6 °C/min to 300 °C (held for seventeen minutes). The concentrations of PCDD/Fs and PCBs were expressed in pg g^−1^ as the sum of 17 PCDD/F and 12 DL-PCB congeners. Cumulative concentrations of PCDD/Fs, DL-PCBs, and PCDD/Fs + PCBs (TEQ_TOT_) were expressed as dioxin toxicity equivalents (WHO TEQ units) using the toxic equivalent factors TEFs: WHO-PCDD/F-TEQ, WHO-PCB-TEQ, and WHO-PCDD/F/PCB-TEQ [28]. Specifically, to have a better risk characterization, we used the TEQ values, which we obtained by the sum of the concentrations of each compound present in the sample multiplied by their TEF values. The TEFs express the toxicity of dioxins, furans, and PCBs in terms of 2,3,7,8-tetrachlorodibenzo-p-dioxin (TCDD), which is considered the most toxic form of dioxin. TEFs for dioxin and dioxin-like compounds are indicated by the World Health Organization (WHO).

The masses under observation were those suggested by the EPA 1613 technique [27]. Using ^13^C_12_-labelled PCDD/F as internal standards, samples were measured using the isotopic dilution method.

#### 2.3.2. Trace Element Analysis

A total of 0.50 g of the sample (honey, bees, and beeswax) was put in a glass test tube and subjected to acid mineralization with 3.0 mL of 70% nitric acid and 1.0 mL of ultrapure water using a Milestone Ultrawave Microwave digestion system (FKW, Torre Boldone, Italy). After the test tubes were allowed to cool to room temperature, the samples were quantitatively recovered and added to 25 mL Class A volumetric flasks using ultrapure water. An Inductively Coupled Plasma Mass Spectrometer (ICP-MS) mod. NexION 3500X (PerkinElmer, Waltham, MA, USA) outfitted with a quartz torch and quartz injector tube (2 mm i.d.) (Glass Expansion, Inc., Melbourne, Australia) was used to determine trace elements. Online mixing was used to introduce rhodium, an internal standard, to the standard and sample solutions at a concentration of 200 ng mL^−1^. Standard solution of arsenic (As), beryllium (Be), bismuth (Bi), cadmium (Cd), cesium (Cs), chromium (Cr), cobalt (Co), iron (Fe), gallium (Ga), indium (In), manganese (Mn), nickel (Ni), lead (Pb), copper (Cu), selenium (Se), strontium (Sr), thallium (Tl), vanadium (V), and zinc (Zn) at 1000 mg/L was obtained from Perkin Elmer (Waltham, MA, USA). Nitric acid 68% (*v*/*v*) was obtained from Romil Ltd. (Cambridge, UK), and high-purity deionized water (resistivity < 18.2 MΩ cm) was produced in-house using an Arium^®^ pro purification system (Sartorius, Göttingen, Germany). Quality assurance and quality control (QA/QC) of the methods for determination of trace elements were monitored by analysis of control samples including procedural blanks, spiked samples, duplicates, and standard solutions measured in each batch.

#### 2.3.3. Polycyclic Aromatic Hydrocarbons Analysis

The methods used for the analysis of PAHs in bee matrices (pollen, bees, honey, and wax) have been reported in previous studies [29,30].

In brief, the extraction was performed using a combination of n-hexane/acetone (1:1, *v*/*v*) from the samples and an accelerated solvent extractor (ASE 100 Dionex Corporation, Sunnyvale, CA, USA). In a rotary evaporator set at 35 °C, the extract was dried after being filtered through filter paper loaded with 3 g of anhydrous sodium sulfate. According to Visciano and colleagues (2008), the extracts were dissolved in 1 mL of acetonitrile and then driven into an HPLC.

A high-performance liquid chromatography (HPLC) device with a 20 μL loop and a variable excitation and emission wavelength fluorescence detector (ProStar 363, Varian, Palo Alto, CA, USA) was used to perform a quantitative study of PAHs. Star Chromatography Workstation version 5.2 (Varian) was the program utilized. All solvents were for trace analysis grade (Carlo Erba Reagents, Val-de-Reuil, France). An EnviroSep PP column (Phenomenex, Torrance, CA, USA; 12.5 cm × 4.60 mm, particle size = 3 μm) and a gradient elution program with a flow rate of 1.4 mL/min were used to separate PAHs at room temperature.

The initial mobile phase was 65% acetonitrile and 35% HPLC water for 8 min, which was then gradually changed to 100% acetonitrile over 1 min, held at 100% for 11 min, and then decreased to the initial phase (65:35%). The investigated PAHs were benzo(a)anthracene (BaA), chrysene (Ch), benzo(b)fluoranthene (BbF), and benzo(a)pyrene (BaP).

The retention time was used to identify PAHs, and an external standard method was used to quantify them. The standard deviation approach was used to calculate the quantification limit (LOQ) and the detection limit (LOD). After injecting a series (*n* = 10) of blank samples in triplicate that had the same matrix as the samples under analysis but without analyte, the mean blank value and standard deviation (SD) were computed. The mean blank value plus 3 SD was the LOD, while the mean blank value plus 6 SD was the LOQ. A minimum of three concentration levels and nine determinations were used to evaluate the method’s accuracy and precision. Analyte recoveries were determined by using honey bees and honey samples spiked with solutions of the PAH standard (PAH-mix9 in acetonitrile) to reach a final concentration of 10, 25, or 50 ng/mL in each sample. The external standard multipoint calibration technique was used to determine the linear response interval of the detector, and the working standard solutions were 1, 5, 10, 25, and 50 ng/mL in acetonitrile.

#### 2.3.4. Pesticides Analysis

Multiresidue analysis of pesticides (Table S2) in hive products was determined using liquid and gas chromatography–tandem mass spectrometry (LC-MS/MS, GC-MS/MS).

Utilizing a QuEChERS kit from Agilent, Santa Clara, CA, USA, the extraction process involved the use of 4.0 g of magnesium sulfate (MgSO_4_), 1.0 g of sodium chloride (NaCl), 1.0 g of sodium citrate, and 0.5 g of sodium citrate sesquihydrate. A kit including 1200 mg of MgSO_4_, 400 mg of primary and secondary amines, and 400 mg of C18 (Agilent, Santa Clara, CA, USA) was used for the cleanup step. At the onset of the extraction process, 0.3 mL of an internal standard solution containing 1 mg/L of triphenylphosphate (TPP, LabService, Italy) was added to all samples except for the matrix blanks. The real samples and the blank samples were handled in the same way.

Well-homogenized samples, including the blanks, were kept in a freezer at −20 °C. These samples served as the basis for both the calibration standards preparation and the fortification testing (recovery measurement). GC-MS/MS and LC/MS/MS were used to directly evaluate the final extract (1.0 mL). The present investigation employed an LC 1290 Infinity II in conjunction with an Agilent 6495 triple quadrupole mass spectrometer to identify pesticides that are susceptible to LC, thus numbering a total of 158 chemicals. The Zorbax Eclipse Plus C18 rapid resolution HD column (150 mm × 2.1 mm × 1.8 μm) (Agilent, Santa Clara, CA, USA) was used to separate all pesticides under study. The mobile phase flow rate was set to 0.40 mL/min, the column oven was maintained at 40 °C, and the autosampler was fixed at 10 °C. There was a 2 μL injection volume. Water (A) and methanol (B) with 5 mM ammonium formate and 0.1% formic acid made up the LC mobile phases.

The following is the elution gradient program used: 0–3 min, 5% eluant B, followed by a linear increase to 100% from 3 to 17 min and a final 3 min of 100% maintenance. The compounds were ionized using the positive ESI + mode. Data processing and acquisition were handled by Agilent MassHunter. A GC System-7010B GC/MS Triple Quadrupole (Agilent Technology, Santa Clara, CA, USA) equipped with a 7693 autosampler and a multimode injector was used to analyze the remaining 75 pesticides. A column HP-5MS (30 m × 0.25 mm × 0.25 μm, Agilent Technology) was fitted to the GC system. The starting column temperature of 60 °C was held for one minute as part of the GC temperature program. After that, the temperature was raised to 120 °C (40 °C/min) and finally to 310 °C at a rate of 5 °C/min. A PTV injector with a 1 μL injection volume was used. The triple quadrupole mass detector has a source temperature of 280 °C and runs in negative EI mode at 70 eV. At a flow rate of 1.5 mL/min, the run took 40.00 min in total, including a 5 min postrun period. Nitrogen (N_2_) was used as the collision gas and helium (He) as the carrier gas, both of which had purity levels of 99.999%. Data processing and acquisition were carried out in MRM mode with MassHunter software (version B.08.00). To obtain matrix-matched calibration curves, pesticide solutions containing 10 mg/L of acetonitrile were acquired from LabService, (Bologna, Italy). The internal standard (TPP) for the pesticides was set at 0.030 mg/L, and five concentration levels (0.005, 0.010, 0.020, 0.050, and 0.100 mg/L) were established. Triphenylphosphate (TPP) was obtained from LabService (Bologna, Italy), and Agilent’s SPE QuEChERS kits were utilized for sample cleaning up. Following the protocols and safeguards put in place to guarantee the dependability of the results by UNI CEI EN ISO/IEC 17025 (2018), quality assurance and quality control (QA/QC) of the analyses were confirmed using control samples, including blanks, spikes, and duplicates. Moreover, participation in interlaboratory research and proficiency tests with z scores consistently falling within the range of ±2 formed part of quality control (QC). To verify the purity of the reagents and rule out any potential laboratory contamination or interference throughout the whole analytical process, blank chemical determinations were performed regularly in conjunction with the tests performed on each batch of samples.

## 3. Results

### 3.1. Dioxins and Dioxin-Like Compounds in Acerra

**Figure 2:**
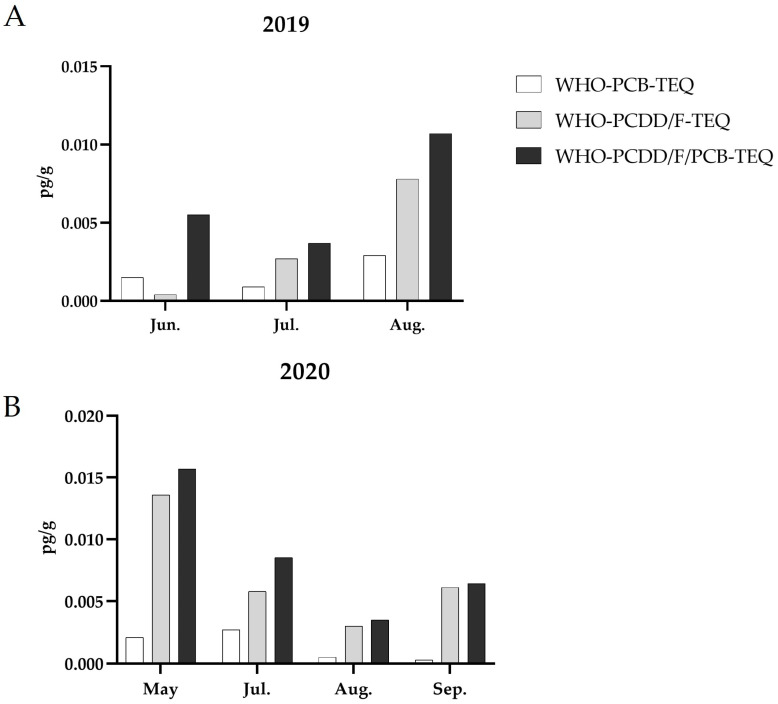
Monthly trends of WHO-TEQ (upper) values of PCBs, PCDD/F, and the sum of PCDD/F/PCB in wax samples collected in Acerra in 2019 (**A**) and 2020 (**B**). Abbreviations: PCB: polychlorinated biphenyls; PCDD/F: polychlorinated dibenzo-p-dioxins and polychlorinated dibenzofurans; Jun: June; Jul.: July; Aug.: August.; Sep.: September.

All the wax samples collected over the years 2019/2020 showed the values of WHO-PCB-TEQ, WHO-PCDD/F-TEQ, and WHO-PCDD/F/PCB-TEQ values below 0.02 pg/g (Figure 2A,B).

The sum of PCBs (WHO-PCB-TEQ) consistently remained below 0.005 pg/g throughout the two years but exhibited two different patterns over time. In 2019, there was a slight increase from June to August, whereas in 2020, the detected levels decreased towards September. Concentrations of PCDD/Fs (WHO-PCDD/F-TEQ) remained below 0.015 pg/g in 2019 but increased in August. In contrast, in 2020, their highest presence was found in the May sample. The sum of all considered species (WHO-PCDD/F/PCB) was higher in the August 2019 and in the May 2020 samples.

### 3.2. Trace Element Analysis

All the samples (honey, bees, and beeswax) collected in 2019 in Acerra had values below the detection limit, except for the Pb values found in the wax samples collected in 2019 ranging from 0.02 to 0.046 mg/kg. In 2020, Pb concentrations found in the Acerra wax had values between 0.016 to 0.217 mg/kg, and the presence of Ni (0.03 mg/kg) and Cr (0.053 mg/kg) was also detected. In the bees collected in 2020, the following metal concentrations were found: Cd—0.019 mg/kg, Cr—0.081 mg/kg, Ni—0.027 mg/kg, and Pb—0.199 mg/kg. In the honey, only Cd and Pb were detected at a concentration of 0.012 mg/kg and 0.041 mg/kg, respectively, in samples from Acerra.

The analyses of metals carried out on bees and wax in the second biennium (2021–2022) in Acerra and Caivano are summarized in Table 1 and Table 2, which show the ranging concentrations found. Regarding the honey, only in a sample collected in Caivano in 2022 did we find a Pb concentration of 0.31 mg/kg.

### 3.3. Polycyclic Aromatic Hydrocarbons

In all hive samples (pollen, bees, wax honey) from Acerra examined during the period 2019–2020, the levels of PAHs were always below the quantification limit (equal to 0.2 µg/kg). In 2021, the total amount of PAHs in the various matrices from Acerra and Caivano did not cause any particular concern, being below the quantification limits. In 2022, the total PAH content resulted above the quantitative limits only in two pollen samples collected in the Acerra and Caivano locations (0.5 µg/kg and 0.65 µg/kg, respectively).

### 3.4. Pesticides

All the samples from Acerra analyzed during the 2019/2020 biennium showed no levels of these substances above the quantification limits, except for one bee sample taken in August 2020, which contained 0.06 mg/kg of the organophosphate pesticide ethyl chlorpyrifos. For the samples obtained in Acerra and Caivano in 2021, the values of nearly 200 pesticides tested were below the limit of quantification, except for dimethoate and piperonyl butoxide, which were detected at quantities of 0.042 and 0.057 mg/kg, respectively, in the Acerra pollen samples. In 2022, in all the tested matrices, pesticides were below the quantification limits, except for azoxystrobin, which was found in a pollen sample from Acerra (collected in September 2022) at a concentration of 0.025 mg/kg.

## 4. Discussion

The use of bees as indicators is not new in the field of biomonitoring, and the installation of apiaries for studying specific areas has also spread in Italy [31,32,33]. In our case, the location of the apiaries makes this study pioneering because, for the first time, the area of interest included a WtE plant and a waste treatment site (S.T.I.R.).

This research was carried out in two stages. The region around Acerra’s WtE plant was the first monitored site through the installation of the apiary and represented the focus of the biennium 2019–2020. By creating a system involving the use of bees as bioindicators, the aim was to evaluate the WtE plant’s safety regarding the release of dioxins and related compounds. The use of bees as a monitoring system is supported by several references in the literature [6,7,32,34,35]; however, to the best of our knowledge, there is little information available regarding the establishment of apiaries close to WtE plants. 

Due to their well-known characteristics of lipophilicity and accumulation, the dioxins PCDD/PCDF and dioxin-like PCBs were primarily investigated in the beeswax samples. Wax functions as a lipidic substrate conducive to the accumulation of lipophilic compounds over time. It acts as a repository for these substances, thus serving as an extracting solvent for liposoluble compounds. Produced by juvenile bees within the hive, wax contamination is associated with the materials—such as nectar, pollen, and the bees themselves—that are conveyed into the hive environment.

Although the limits of dioxins in apiary products are not set in Regulation EU 2023/915 relating to maximum levels of certain contaminants in foods, their levels in 2019–2020 resulted below the precautionary limits imposed on foods intended for infants, where the sum of dioxins (WHO-PCDD/F-TEQ/g) and dioxin-like PCBs have been set at 0.1 pg/g and 0.2 pg/g wet weight, respectively. We were unable to identify the specific sources of pollution as a consequence of the bees’ approximately 3 km foraging radius and the numerous sources of pollutants in that area. However, the results offered a comprehensive view, thereby allowing us to formulate hypotheses about the influence of external factors on pollutant contamination. For example, we observed a trend toward decreasing levels of dioxins PCDD/PCDF and dioxin-like PCBs and related compounds around 2020, which could be attributed to the reduction in human activities due to COVID-19 restrictions during the lockdown period [36]. To assess the role of human activities in the production of dioxins, it is interesting to compare these data with those from areas characterized by a heavy industrial influence, such as the Bologna central station area [37], where an apiary monitoring station was installed inside Ducati Motor Holding S.p.A.s’ headquarters. In that study, a greater amount of PCDD/Fs was found in the wax (the average WHO-TEQ (upper bound) value measured on the average PCDD/PCDF concentrations from 2017 samples was around 0.22 pg/g).

Because of the possible harmful effects and illnesses that heavy metal pollution may cause in humans [38] as well as its impacts on ecosystem stability, during the biennium 2019–2020, bees, wax, and honey from Acerra were tested for metals deriving from anthropogenic sources. Cr was indicative of the presence of chrome plating industries, while the sources of Ni and Cd could be different (industrial processes, vehicle emissions, combustion of fossil fuels, waste disposal, or the use of fertilizers and pesticides in agricultural practices). Regarding Pb, it certainly deserved particular attention. It has been used extensively in the manufacturing of batteries, alloys, paints, enamels, water distribution systems, automobile gasoline, and aviation fuel, among other consumer goods. Even though many of these uses are currently forbidden (at least in Europe), Pb is still present in many products and monitored for related health issues [39]. Although there is currently no dedicated legislative framework for beeswax, the data obtained in 2019–2020 can be crossreferenced with the specifications outlined in European Regulation 915/2023, which delineates the maximum permissible concentrations of select contaminants in food. The prescribed limits for Pb range from 0.02 mg/kg for infant food products to 3 mg/kg for dietary supplements.

During the second phase of the study, the data were also gathered at the Caivano site, thus allowing for a comparative analysis between the two monitoring areas for the biennium 2021–2022. More elevated ranges of the selected metals Cu, Fe, Mn, and Zn were observed in Caivano. These discrepancies between Acerra and Caivano could be attributed to geological disparities and land use patterns. The Caivano area is distinguished by a higher concentration of industries and traffic. Moreover, fluctuations were also observed based on the sampling year, with Fe ranges tending to decrease in 2021 in both sites. These observations demonstrated the sensitivity of this bioindicator to external variations, such as the suspension of certain anthropogenic activities due to the COVID-19 pandemic. This aligns with findings reported by Scivicco et al. (2022), who documented the reduction of metal levels in bees during the lockdown in recent research conducted in the Campania region (2022) [40].

Because honey is a significant food product for humans, it merits special attention. In compliance with Italian legislation (Legislative Decree no. 179/2004, which implements Directive 2001/110/EC on honey production and commercialization), analyses were carried out on it. Specific references to allowable limits of potentially hazardous substances in honey and other apiculture products are scarce in European directives. The only metal for which honey has maximum values is Pb, which, per EU Regulation 915/2023, cannot be more than 0.10 mg/kg. Therefore, the honey from Acerra was found to be of good food quality in 2019–2020. The Pb concentration found in 2022 honey from Caivano demonstrated that the resumption of human activities may influence the quality of apiary products [14,41].

Bees can capture an instantaneous picture of the state of the environment. This is especially true about PAHs, and their levels clearly showed a decline during the period of reduced anthropogenic activities (up to 2021) and then showed a slight increase in 2022, which corresponded with the start of human activity again. Comparing the Caivano to the Acerra area, it is clear that there is significantly more vehicle traffic there. The Caivano area, in addition to industries, is very close to high-speed train tracks.

Regarding the pesticides, results showed the presence of the organophosphate ethyl chlorpyrifos in one bee sample from Acerra (2020), but this detection might be related to its sporadic use on crops. In 2021, dimethoate and piperonyl butoxide were found in pollen collected in Acerra. Dimethoate has been used to control pests and is frequently applied to crops to keep off aphids, thrips, mites, leafhoppers, whiteflies, and caterpillars [42]. Although it has not recently had its approval renewed in Europe, dimethoate has continued to be found in food, thereby suggesting an illegal use. Pollen for honey and other apiculture products have no special recommendations about dimethoate limits (Reg. (EU) 2021/155), and given that some categories lack absolute references, we can only compare the measured concentrations with those listed in the FAO/WHO Codex Alimentarius and EU Regulation 2021/155, where the limit range is between 0.01 to 0.05 mg/L. Piperonyl butoxide was also found in pollen samples from Acerra (2021). It is utilized in a variety of insecticide and pesticide formulations as a synergist. Compared with the data reported by the codex Alimentarius, the detected concentrations fall within the range of the maximum residue levels (https://www.fao.org/fao-who-codexalimentarius/codex-texts/dbs/pestres/pesticide-detail/en/?p_id=62 (accessed on 3 March 2019)). In pollen samples from Acerra (2022), azoxystrobin presence was found. It is a broad spectrum systemic fungicide widely used in agriculture to protect against fungal diseases. There are no particularly detailed studies on this molecule, although recently it was shown to have sublethal effects in the midgut of the honey bees [43]. In this study, the concentration detected was well below the limits proposed by the European Food Safety Authority (EFSA). Beyond the possible reasons for these pesticides’ sporadic presence, the data demonstrate the value of bees—and in this case, the related matrix pollen—in determining the kind of anthropogenic activity that best refers to a particular location. Under this scenario, the Acerra region’s predominant agricultural activity could be distinguished from the Caivano territory’s more prominent industrial sector by the presence of pesticides, which were difficult to detect in the latter.

## 5. Conclusions

These results are based on a preliminary study and will require additional observations. Still, it certainly starts an inquiry about the fascinating application of the bee as a bioindicator for tracking areas of specific interest, like those close to urban waste treatment plants. *Apis mellifera* presents several advantages over alternative models, notably in terms of its ease of management and accessibility to diverse matrices, including pollen, wax, and honey. However, careful consideration should be given to the selection of appropriate substrates to effectively identify pollutants with specific physicochemical characteristics, such as lipophilicity or hydrophilicity.

In our investigation, the findings highlighted the effectiveness of the Acerra WtE plant in mitigating the production and release of hazardous species, particularly dioxins. Moreover, bees emerged as versatile tools for detecting environmental contaminants, including pesticides, PAHs, and heavy metals. The installation of a secondary monitoring site in Caivano provided additional insights into the environmental quality proximal to the Waste Shredding, Sifting, and Packaging Plant, thus demonstrating the feasibility of comparing two adjacent critical areas (Acerra and Caivano). Notably, these bioindicators demonstrated sensitivity to changes in the external environment, such as the reduction in anthropogenic activities during the 2020–2021 lockdown period.

Regular environmental evaluations conducted using bees demonstrated the ability to look into contaminant fluxes, which hold promise for improving public health protection. Additionally, this study suggests the necessity of broader and more precisely defined regulatory structures controlling the existence of pollutants in beekeeping goods. Such measures are necessary to establish reference thresholds for permissible concentrations of distinct pollutants.

## Figures and Tables

**Table 1:** Ranges of metal concentrations (mg/kg) found in bees across the specified years and sites of sampling.

Year	Metal	Site	
2021		Acerra	Caivano
	Cu	6.19–8.03	6.94–9.57
	Fe	34.2–43.1	39.4–54.7
	Mn	11.3–18.0	27.6–20.3
	Zn	23.7–22.7	22.4–32.4
2022	Cu	0.34–8.26	7.37–8.07
	Fe	40.5–59.9	43.1–83.7
	Mn	9.4–24.8	9.71–22.9
	Zn	21.6–27.7	24.8–28.3

**Table 2:** Ranges of metal concentrations (mg/kg) found in wax across the specified years, months, and sites of sampling.

Year	Metal	Site	
2021 *		Acerra	Caivano
	Cu	0.19	0.91
	Fe	2.02	6.72
	Mn	0.08	0.50
	Zn	4.12	5.21
2022	Cu	0.27–0.39	0.56–1.07
	Fe	2.64–10.6	11.5–13.3
	Mn	0.61–0.66	0.36–0.84
	Zn	0.74–16.0	4.67–9.31

* Only one sampling was possible in 2021.

## Data Availability

The data presented in this study are available on request from the corresponding author. The data are not publicly available due to privacy restrictions.

## Supplementary Materials

The following supporting information can be downloaded at: https://www.mdpi.com/article/10.3390/ani14101446/s1, Table S1: Dioxins and dioxin-related compounds; Table S2: Pesticides.
