# Supplementary material for: Use of Honey Bees and Hive Products as Bioindicators to Assess Environmental Contamination in Targeted Areas of the Campania Region (Italy)
Source: Animals (Basel). 2024 May 13;14(10):1446. doi: 10.3390/ani14101446 (PMC11117287; doi:10.3390/ani14101446)
Supplement: Supplementary file 1 [file animals-14-01446-s001.zip › animals-2940762-SI.pdf]

Supplementary

Table S1-Dioxins and dioxin-related compounds

Dioxins
1,2,3,4,5,6,7,8 OCDF
1,2,3,4,6,7,8,9 OCDD
1,2,3,4,6,7,8 HPCDD
1,2,3,4,6,7,8 HPCDF
1,2,3,4,7,8,9- HPCDF
1,2,3,4,7,8,- HXCDD
1,2,3,4,7,8,- HXCDF
1,2,3,6,7,8,- HXCDD
1,2,3,6,7,8,- HXCDF
1,2,3,7,8,9- HXCDD
1,2,3,7,8,9- HXCDF
1,2,3,7,8-PEC DD
1,2,3,7,8-PEC DF
2,3,4,6,7,8 HXCDF
2,3,4,7,8 PEC DF
2,3,7,8 TCDD
2,3,7,8 TCDF

PCBs-DL
77
81
169
105
114
118
123
126
156
157
167
189
PCBs-NdL
28
52
101

153
138
180

Table S2-Pesticides

Pesticides
Acephate
Acetamiprid
Aldicarb fragment
Aldicarb sulfone
Aldicarb sulfoxide
Ametryn
Aminocarb
Amitraz
Atrazine
Atrazine-desethyl
Azaconazole
Azinphos-methyl _ Guthion
Azoxystrobin
Bendiocarb
Benzoximate
Bitertanol
Boscalid (Nicobifen)
Bromoxynil
Buprofezin
Butocarboxim
Carbaryl
Carbophenothion methyl
Carfentrazone-ethyl
Chlorfenvinphos
Chlorfluazuron
Chlorotoluron
Clofentezin
Clomazone
Clothianidin
Coumaphos
Cyazofamid
Cycluron
Cyflufenamid
Cymoxanil

Cyproconazole
Cyprodinil
Cyromazine
Demeton-S-methyl
Demeton-s-methyl-sulfone
Demeton-s-methyl-sulfoxide
Dicrotophos
Diethofencarb
Difenoconazole
Diflubenzuron
Diflufenican
Dimethoate
Dimethomorph
Dimoxystrobin
Diniconazole
Diuron
Epoxyconazol
Ethiofencarb
Ethion
Ethirimol
Etoxazole
Famoxadone
Fenamidon
Fenamiphos
Fenamiphos sulfone
Fenamiphos sulfoxide
Fenarimol
Fenazaquin
Fenbuconazole
Fenchlorphos oxon
Fenoxycarb
Fenpropidin
Fenpropimorph
Fenpyroximat
Fenuron
Fluazinam
Flubendiamide
Fludioxonil
Fluopicolide
Fluquinconazole
Flusilazole
Flutriafol

Forchlorfenuron
Fosthiazate
Fuberidazol
Furalaxyl
Imazalil
Imidacloprid
Indoxacarb
Ioxynil
Iprovalicarb
Isoproturon
Isoxaben
Kresoxim-methyl
Lenacil
Linuron
Malaoxon
Malathion
Mandipropamid
Mecarbam
Mepanipyrim
Mepronil
Metalaxyl
Metamitron
Metconazole
Methabenzthiazuron
Methamidophos
Methiocarb
Methiocarb sulfone
Methiocarb sulfoxide
Methomyl
Methoxyfenozide
Metobromuron
Metoxuron
Metrafenon
Metribuzin
Mevinphos
Monocrotophos
Monolinuron
Myclobutanil
Napropamide
Oxadiazon
Oxadixyl
Paclobutrazol
Penconazole
Pencycuron

Pendimethalin
Phosphamidon
Phoxim
Picolinafen
Picoxystrobin
Piperonyl butoxide
Pirimifos-ethyl
Profenofos
Promecarb
Prometon
Prometryn
Propamocarb
Propaquizafop
Propiconazole
Propoxur
Propyzamide (Pronamide)
Proquinazid
Pyridaben
Quinalphos
Quinoxyfen
Quizalofop-ethyl
Silthiofam
Simazine
Simetryn
Spinosad A+D
Spirodiclofen
Spiroxamine
Tebuconazole
Tebufenozid
Tebufenpyrad
Terbumeton
Tetraconazole
Thiabendazol
Thiacloprid
Thiamethoxam
Triadimefon
Triazophos
Tribenuron-methyl
Tricyclazol
Trifloxystrobin
Triflumuron
Vamidothion
Zoxamide

2,4 DDD
2,4 DDE
2,4 DDT
4,4 DDD
4,4 DDE
4,4 DDT
Acetochlor
Alachlor
Aldrin
Benfluralin
Bifenthrine
Biphenyl
Bromocyclen
Bromophos ethyl
Chlofenapyr
Chlordane cis
Chlordane trans
Chlorfenviphos
Chloropropilate
Chlorprofam
Chlorpyriphos ethyl
Chlorpyriphos methyl
Crimidine
Cyanophos
Cymoxanil
Cypermethrin
Deltamethrin
Desmetryn
Diazinon
Dichlofenthion
Dieldrin
Dimepiperate
Dipropetryn
ECB
Endosulfan alfa
Endosulfan beta
Endosulfan sulfate
Etaconazole
Etrimfos
Famphur
Fenchlorphos (Ronnel)
Fenitrotion
Fenpropathrin
Fipronil

Fipronil desulinyl
Fipronil sulfone
Fonofos
HCH agamma (lindane)
HCH alfa
HCH beta
Heptachlor
Heptachlor epoxide cis
Heptachlor epoxide trans
Heptenophos
Hexaconazole
Isodrin
Nuarimol
Ofurace
Oxyfluorfen
Parathion ethyl
Permethrine cis
Permethrine trans
Pirimicarb
Pirimiphos methyl
Propachlor
Propanil
Sulfotep
Sulprofos
Tecnazene
Tefluthrin
Terbutryn
Tetradifon
Trichloronat
Vinclozolin
